# Dietary carotenoid availability, sexual signalling and functional fertility in sticklebacks

**DOI:** 10.1098/rsbl.2009.0815

**Published:** 2009-11-18

**Authors:** Thomas W. Pike, Jonathan D. Blount, Jan Lindström, Neil B. Metcalfe

**Affiliations:** 1 Division of Ecology and Evolutionary Biology, Faculty of Biomedical and Life Sciences, University of Glasgow, Graham Kerr Building, Glasgow G12 8QQ, UK; 2 Centre for Ecology and Conservation, School of Biosciences, University of Exeter, Cornwall Campus, Penryn TR10 9EZ, UK

**Keywords:** fertility, carotenoid coloration, antioxidants, oxidative stress

## Abstract

In species where males express carotenoid-based sexual signals, more intensely coloured males may be signalling their enhanced ability to combat oxidative stress. This may include mitigating deleterious oxidative damage to their sperm, and so be directly related to their functional fertility. Using a split-clutch *in vitro* fertilization technique and dietary carotenoid manipulation, we demonstrate that in non-competitive fertilization assays, male three-spined sticklebacks (*Gasterosteus aculeatus*) that are fed higher (but biologically relevant) levels of carotenoids had a significantly increased fertilization success, irrespective of maternal carotenoid intake. Furthermore, within diet groups, a male's fertilization success was positively related to the expression of his carotenoid-based nuptial coloration, with more intensely coloured males having higher functional fertility. These data provide, to our knowledge, the first demonstration that dietary access to carotenoids influences fertilization success, and suggest that females could use a male's nuptial coloration as an indicator of his functional fertility.

## 1. Introduction

It has been hypothesized that a male's secondary sexual ornamentation may provide females with cues about his functional fertility (the success of ejaculates in fertilizing eggs; Sheldon 1994). This could potentially explain the maintenance of female preference for male ornaments as a result of direct selection in non-resource-based mating systems, where the indirect benefits have remained elusive (Kotiaho & Puurtinen 2007). A number of studies have presented correlative evidence for a link between secondary sexual signals and functional fertility (or, as a proxy, sperm characteristics) in various taxa (e.g. Malo *et al*. 2005; Pitcher *et al*. 2007; Polak & Simmons 2009). However, direct evidence linking functional fertility and ornamentation is lacking, although a number of potential physiological explanations for such a link have been proposed. Here, we test the hypothesis (Blount *et al*. 2001; Velando *et al*. 2008) that antioxidant availability may be limited for the expression of sexual ornaments and the maintenance of fertility; if antioxidants are limited, allocation of antioxidants to sexual signals may trade off against antioxidant protection of developing gametes, an important factor affecting DNA damage in the male germ line (Lewis & Aitken 2005; Olsen *et al*. 2005; reviewed in Velando *et al*. 2008). Carotenoids, with their dual role as both antioxidants and pigments involved in the formation of many sexual ornaments (Krinsky 1998; Olson & Owens 1998; von Schantz *et al*. 1999), have been proposed to play a key role in this process (Blount *et al*. 2001), leading us to predict a direct association between dietary carotenoid availability and functional fertility.

We tested this predication using a split-clutch *in vitro* fertilization (IVF) technique (Barber & Arnott 2000) in three-spined sticklebacks (*Gasterosteus aculeatus*). During the breeding season, male sticklebacks develop a region of red nuptial coloration based on carotenoids that are limited in the diet (Barber *et al*. 2000), and in mate-choice trials females generally prefer males expressing more intense, carotenoid-rich coloration (Wootton 1984). Males obtaining lower levels of carotenoids in the diet cannot maintain their red coloration over the breeding season and show a significantly higher susceptibility to oxidative stress, suggesting that the carotenoid coloration is a signal of somatic antioxidant defences (Pike *et al*. 2007*a*; Lindström *et al*. 2009).

## 2. Material and methods

Juvenile three-spined sticklebacks were reared until sexual maturation on a diet based on antioxidant-free fish-feed pellets supplemented with either high (200 µg g^−1^) or low (10 µg g^−1^) levels of carotenoids (astaxanthin, lutein and zeaxanthin), but which were otherwise nutritionally identical. Rearing conditions and diet preparation were as described in Pike *et al*. (2007*a*). Twenty sexually mature males from each diet treatment were provided with nesting material and induced to build nests (Pike *et al*. 2007*a*,*b*). After nest building was complete, a body condition index was calculated as mass/length^3^ (Bolger & Connolly 1989) and standardized reflectance scans of their nuptial coloration were taken (Pike *et al*. 2007*b*). These were used to calculate signal chroma (colour saturation; Pike *et al*. 2007*b*), which is known to predict the concentration of carotenoids that an individual invests in sexual ornamentation (*R*^2^ = 0.61; T. W. Pike 2005, unpublished data from biochemical analysis) and mating success (Pike *et al*. 2007*b*).

Following reflectance measurements, males were immediately killed with an overdose of anaesthesia (benzocaine) and dissected to remove the testes and seminal vesicles, which were transferred to sterilized watch glasses and macerated using fine forceps in 1 ml aerated distilled water. Males were processed in a random order with respect to diet treatment. Eggs were obtained from a sample of gravid females (*n* = 10 from each diet treatment) that had recently given a ‘head-up’ response to a courting male (an indication of their readiness to spawn; Wootton 1984) by gently squeezing the abdomen. Each clutch was then carefully divided into two approximately equal halves (mean ± s.e.: 46.6 ± 8.5 eggs in each half; paired *t*-test: *t*_19_ = 0.83, *p* = 0.42) using fine tweezers and each half transferred into separate watch glasses, one containing sperm from a randomly selected male on a high-carotenoid diet and the other containing sperm from a randomly selected low-carotenoid diet male. Sperm and eggs were mixed by gentle agitation for 5 min and then left to stand for 15 min at room temperature (*ca* 18°C) (Barber & Arnott 2000; Bakker *et al*. 2006).

Egg masses were then transferred to plastic containers (200 ml) filled with aerated matured water, to which we added a small quantity of methylene blue (2.5 µg ml^−1^) to reduce fungal infection (Barber & Arnott 2000); they were then incubated for 24 h at 15 ± 1°C. The total number of eggs in each half clutch and the number of these that were fertilized (Swarup 1958) were then counted using a binocular microscope (Bakker *et al*. 2006). Functional fertility was defined as the proportion of fertilized eggs (Bakker *et al*. 2006).

Testes were extracted from a further sample of males (*n* = 10 from each diet treatment), and tested for total antioxidant activity using the Trolox equivalent antioxidant capacity (TEAC) assay (Rice-Evans & Miller 1994). Higher scores indicate that an individual has a greater total antioxidant capacity (measured in millimoles of Trolox equivalents per length).

Complete methodological details are provided in the electronic supplementary material. The experiment was performed under licence from the UK Home Office.

## 3. Results

**Figure 1.**
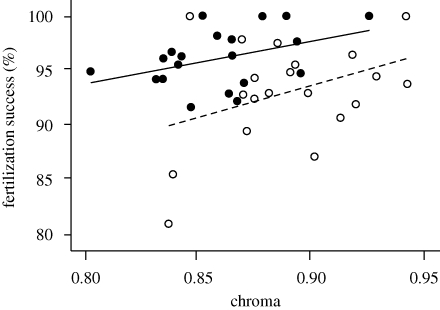
The relationship between sexual signal chroma and functional fertility (percentage of fertilized eggs) in males on high- (black points, solid line) and low-carotenoid (white points, dashed line) diet treatments. Least-squares regression lines are shown.

Male sticklebacks fed with high levels of carotenoids had a significantly higher functional fertility than those fed with lower carotenoid levels (linear mixed-effects model, *F*_1,17_ = 6.73, *p* = 0.019; figure 1), independent of maternal carotenoid intake (*F*_1,17_ = 0.99, *p* = 0.33) or male body condition (*F*_1,17_ = 0.02, *p* = 0.90). Moreover, the chroma of a male's sexual ornamentation was significantly positively related to functional fertility (*F*_1,17_ = 7.03, *p* = 0.017; figure 1), with males expressing more intense regions of nuptial coloration, in both diet treatment groups, fertilizing a greater proportion of eggs. Chroma also differed significantly between males on the two diet treatments (two-sample *t*-test, *t*_38_ = 3.21, *p* = 0.003); with low-carotenoid diet males actually developing more intense coloration than males on the high-carotenoid diet (figure 1). Body condition did not differ between treatment groups (mean ± s.e.: high, 14.1 ± 1.14; low, 12.8 ± 0.95; two-sample *t*-test, *t*_38_ = 0.91, *p* = 0.37).

The testes of high-carotenoid diet males had a significantly greater antioxidant capacity than those of males on the low-carotenoid diet (high: 0.12 ± 0.02 mmol l^−1^; low: 0.06 ± 0.01 mmol l^−1^; *t*_18_ = 2.46, *p* = 0.024).

## 4. Discussion

The finding that males with a lower intake of carotenoids actually developed the more intense nuptial coloration might seem counterintuitive, but is actually in line with theoretical predictions: we have recently shown that stickleback males in poor condition should prioritize their investment in sexual ornaments at the start of the breeding season (as was the case in the present experiment), even at the expense of future survival, as this will maximize their chances of reproducing (Lindström *et al*. 2009). It is also consistent with the finding that breeding males on the low-carotenoid diet put a far greater proportion of their total body carotenoids into their throat patch than males on a high-carotenoid diet, but accumulate more oxidative damage and die sooner as a result (Pike *et al*. 2007*a*). The present study shows that this was also associated with an effect on male functional fertility, where the half of a female's egg mass fertilized by males from the high-carotenoid diet treatment contained a significantly higher proportion of fertilized eggs than the half fertilized by low-carotenoid diet males.

There is considerable evidence in humans and domesticated species that high levels of oxidative stress associated with low availability of antioxidants can cause damage to sperm and hence reduce fertility, and that these effects can be mitigated by antioxidants (reviewed in Velando *et al*. 2008). Given that our dietary manipulation created corresponding differences in oxidative stress in the somatic tissues of the males (Pike *et al*. 2007*a*), a likely mechanism for the difference in fertilization success between diet treatments is that the sperm of low-carotenoid diet males was subjected to increased oxidative damage (Blount *et al*. 2001; Velando *et al*. 2008). This is supported by the finding that the sperm of low-carotenoid diet males had a significantly lower antioxidant capacity (i.e. poorer reserves of functional antioxidants), and therefore impaired potential to scavenge reactive oxygen species (ROS) and mitigate the negative effects of oxidative stress (Velando *et al*. 2008). Alternatively, there could have been differences in the sperm production of the treatment groups, affecting the males’ ability to fertilize a batch of eggs. Both mechanisms could affect functional fertility, but remain to be verified physiologically.

The relationship between signal intensity and functional fertility was not negative, as might be expected if there was a simple trade-off between the two traits, but positive in both diet treatment groups. This finding is consistent with the results of a recent correlative study by Peters *et al*. (2004), who demonstrated positive associations between carotenoid-based bill coloration and sperm velocity in mallards (*Anas platyrhynchos*) (but see Pitcher *et al*. 2009), and might be explained by variation among individuals in their acquisition and requirement for resources, which can generate positive as well as negative correlations among life-history traits (Van Noordwijk & de Jong 1986). For instance, males within a diet treatment group might vary in their ability to assimilate antioxidants from the diet (which may be considered an aspect of male ‘quality’). Similarly, if individual sticklebacks vary in their investment in reproduction in accordance with their ability to deal with the costs of such investment (i.e. individual optimization), tests for trade-offs using cross-sectional data will typically yield correlations in the opposite direction to those predicted by life-history theory (e.g. Hamel *et al*. 2009). Furthermore, honest signalling need not be a handicap (Getty 1998), and individuals with superior ability to acquire or assimilate dietary carotenoids may be able to simultaneously increase both nuptial coloration and functional fertility. The fact that males of poor quality, or in poorer condition as affected by carotenoid availability, cannot sustain their red coloration during the breeding season (Pike *et al*. 2007*a*; Lindström *et al*. 2009) suggests that, as time progresses, negative correlations between nuptial coloration and functional fertility may arise.

Female sticklebacks from most populations, including our own (Pike *et al*. 2007*a*), show a preference for redder males (Wootton 1984). Our data provide, to our knowledge, the first demonstration that dietary access to carotenoids influences functional fertility, consistent with Blount *et al*.'s (2001) hypothesis. Therefore, by selecting males with the most intense nuptial coloration, females may be able to select for mates with the greatest functional fertility (cf. Sheldon 1994).
